# Supplementary material for: Profiling Dynamic Patterns of Single‐Cell Motility
Source: Adv Sci (Weinh). 2024 Aug 13;11(38):2400918. doi: 10.1002/advs.202400918 (PMC11481225; doi:10.1002/advs.202400918)
Supplement: Supplementary file 1 — Supporting Information [file ADVS-11-2400918-s003.pdf]

ADVANCED SCIENCE
Open Access

Supporting Information

for *Adv. Sci.*, DOI 10.1002/advs.202400918

Profiling Dynamic Patterns of Single-Cell Motility

*Debonil Maity, Nikita Sivakumar, Pratik Kamat, Nahuel Zamponi, Chanhong Min, Wenxuan Du, Hasini Jayatilaka, Adrian Johnston, Bartholomew Starich, Anshika Agrawal, Deanna Riley, Leandro Venturutti, Ari Melnick, Leandro Cerchietti, Jeremy Walston and Jude M. Phillip**

SUPPORTING INFORMATION

Title: Profiling Dynamic Patterns of Single-cell Motility

Authors: Debonil Maity*, Nikita Sivakumar*, Pratik Kamat, Nahuel Zamponi, Chanhong Min, Wenxuan Du, Hasini Jayatilaka, Adrian Johnston, Bartholomew Starich, Anshika Agrawal, Deanna Riley, Leandro Venturutti, Ari Melnick, Leandro Cerchietti, Jeremy Walston, Jude M. Phillip

---

Figure S1. Data and harmonization across multiple datasets. A. Trajectory of a single ht1080 cell showing the effects of varying interval times (2 to 10 minutes per 30-second steps). Box and whisker plot below show the magnitude of the geometric concordance with varying intervals. **B**. Trajectory of an ht1080 cell at a time resolution of 4 minutes (blue) and the predicted 4-minute intervals based on interpolation (purple) and pseudo-Monte Carlo method (pMC-2.01, brown). Box and whisker plot below show the magnitude of the geometric concordance. **C**. Trajectory of an ht1080 cell at a time resolution of 6 minutes (blue) and the predicted 6-minute intervals based on interpolation (purple) and pseudo-Monte Carlo method (pMC-2.01, brown). Box and whisker plot below show the magnitude of the geometric concordance. In both cases (B and C), the starting time resolution was 8 minutes.

Figure S2. Sensitivity analysis across pMC factors. A. Mean squared displacements (MSD) of primary human dermal fibroblasts (n=873 cells) as a function of time lag. Data indicates that interpolation and pMC1 exhibits significant deviations relative to original trajectories at short time-scales. **B**. Sensitivity analysis showing comparisons in cell motility parameters for original, interpolation, and four pMC factors.

Figure S3. Characterization of short duration cell motility clusters. A. Communality factor analysis for the use of eight APRW motility parameters. Bar plots show the average communality score for eight APRW-based parameters relative to other base motility features (Heteromotility, Kimmel et al. PLOS Computational Biology, 2018). **B**. Line plot shows the number of clusters and the inertia/silhouette ratios. **C**. Heatmap of cluster-coherence showing that cells classified within each motility cluster were close to their cluster centroids. The characteristic 'staircase' pattern indicates that cells clustered within each group exhibit short distances to the centroid of the respective clusters. **D**. Radar plot showing the magnitude of the eight motility parameters for the seventeen short duration motility clusters. Magnitude of the motility parameters per cluster highlight some of the fundamental differences among clusters.

Figure S4. Comparison of higher order parameter distributions between interpolated (orange) and original (blue) trajectories for 17 short-duration motility clusters. To understand whether pMC interpolation introduces systematic artifacts in higher order parameter values that impact clustering analysis, we compared the higher order parameter distributions for a random sample of original trajectories (blue) and their pMC interpolation counterparts (orange). The distributions tend to follow the same shape. Black bars indicate the 95% confidence interval for the mean, which overlap between original and interpolated trajectories.

Figure S5. T-cell lymphoma motility in response to microtubule perturbation. (A) Heatmap indicating normalized higher order motility parameter values for T-cell lymphoma cells exposed to varying concentrations of Taxol and Vincristine (Vinc). **(B)** Distribution of 17 short-duration motility clusters for T-cell lymphoma cells exposed to varying concentration of Taxol and Vinc. **(C)** 2D tSNE representation of multi-dimensional motility distribution for each condition on the tSNE space constructed previously.
